# Compliance with COVID-19 Physical Distancing Mandates in Oman: The Role of Health Literacy and Internal Health Locus of Control

**DOI:** 10.3928/24748307-20240424-01

**Published:** 2024-04

**Authors:** Amna Alabri

## Abstract

**Background:**

Research indicates that the effectiveness of coronavirus disease 2019 (COVID-19) physical distancing mandates is influenced by several individual factors, including health literacy; internal health locus of control (IHLOC), the belief that physical distancing can reduce COVID-19 risk; social norms; self-efficacy; and perceptions of the benefits and barriers associated with distancing. However, further investigation is needed to understand the links between these factors and compliance intentions.

**Objective:**

This study investigates the mechanism linking these factors with the intentions to comply with physical distancing mandates.

**Methods:**

A total of 759 participants (Mean age = 29.13, standard deviation [*SD*] = 8.33; 68.5% women) were surveyed online from September 2020 to October 2020. Data were analyzed using ANOVA (analysis of variance) and structural equation modeling.

**Key Results:**

Health literacy was associated with more perceived benefits (*β* = .175, *p* = .001), greater self-efficacy (*β* = .193, *p* < .001), and less perceived barriers (β = −.391, *p* < .001). IHLOC was significantly associated with greater perceived benefits (*β* = .156, *p* = .007) and self-efficacy (*β* = .294, *p* < .001). Family descriptive norms were significantly associated with fewer perceived barriers (β = −.276, *p* < .001), while injunctive norms were associated with more perceived benefits (*β* = .202, *p* = .001) and higher self-efficacy (*β* = .299, *p* < .001). Intentions to adhere to physical distancing mandates were significantly associated with past compliance (*β* = .427, *p* < .001) and perceived barriers (β = −.205, *p* < .001) and benefits (*β* = .295, *p* < .001). Post-hoc mediation analyses revealed several small yet significant indirect effects, highlighting the complex pathways shaping adherence intentions.

**Conclusions:**

This study identifies how health literacy, IHLOC, social norms, perceived benefits and barriers, and self-efficacy intricately shape intentions to comply with physical distancing mandates. These findings offer valuable implications for public health policy and interventions. [***HLRP: Health Literacy Research and Practice*. 2024;8(2):e69–e78.**]

## Abstract (plain language summary)

**Plain Language Summary: **This study examined the factors associated with Omanis' intentions to comply with physical distancing mandates during COVID-19. Results revealed that individuals with higher health literacy perceived fewer barriers and more benefits to physical distancing, making them more willing to comply with mandates. Those who believed their actions could reduce the risk of contracting the virus also reported greater benefits and were more likely to comply.

Coronavirus disease 2019 (COVID-19) drastically altered daily life, necessitating significant reductions in social interactions to curtail the virus's spread (Flaxman et al., 2020; Guo et al., 2021). Governments worldwide swiftly implemented physical (i.e., social) distancing policies to delay and flatten the pandemic curve (European Centre for Disease Prevention and Control, 2020). These measures compelled individuals to forgo routine activities, like social gatherings, and adopt new norms, such as mask-wearing. Numerous studies confirm the effectiveness of distancing in pandemic control (Chen et al., 2020; Chung & Chan, 2021; Matrajt & Leung, 2020; Ricoca Peixoto et al., 2020). For instance, had China adopted distancing measures 1 to 3 weeks sooner, case counts might have been reduced by 66% to 95%, and the virus's geographic reach could have been substantially limited (Lai et al., 2020).

The effectiveness of physical distancing mandates is influenced by policy enforcement (Chung & Chan, 2021), cultural norms (Cho & Lee, 2015; Kapitány-Fövény & Sulyok, 2020), and individual factors that shape individuals' interpretation, perception, and response to these mandates. Factors such as health literacy, internal health locus of control (IHLOC), social norms, self-efficacy, and perceptions of benefits and barriers influence health-related decisions (Bennett et al., 2017; Fishbein & Ajzen, 2005; Hamilton et al., 2020; Nutbeam et al., 2018; Paasche-Orlow & Wolf, 2007; Steinmetz et al., 2016).

Health literacy is the ability to access, acquire, navigate, process, and understand health-related information and services to make informed health decisions (Nutbeam et al., 2018; Paasche-Orlow & Wolf, 2007). People with low health literacy struggle to comprehend health risks and complex health-related information, leading to adverse health outcomes (Berkman et al., 2011; Diviani et al., 2015; Goto et al., 2019; Kim & Han, 2016; Oldach & Katz, 2014; Sarma et al., 2019). During the COVID-19 pandemic, health literacy has emerged as a crucial determinant of health outcomes and disparities. Low health literacy has been linked to a poor understanding of COVID-19 symptoms and preventive measures (James et al., 2022; McCaffery et al., 2020; Rodon et al., 2022). Additionally, individuals with limited health literacy were more prone to endorsing COVID-19 misinformation and underestimating the value of physical distancing (McCaffery et al., 2020; Riiser et al., 2020).

IHLOC refers to individuals' beliefs about how their actions influence health outcomes (Steptoe & Wardle, 2001). Those with a high IHLOC are more likely to believe that behaviors such as maintaining a healthy diet, engaging in regular exercise, or adopting preventive measures are crucial in determining their health status. In contrast, those with an external health locus of control often attribute health outcomes to factors outside their control, such as luck or fate. IHLOC typically motivates individuals to adopt preventive and healthier behaviors by attributing health outcomes to personal actions (Bennett et al., 2017; Chen et al., 2010; Grotz et al., 2011; Marr & Wilcox, 2015; Roncancio et al., 2012). During the COVID-19 pandemic, those with higher IHLOC showed heightened self-efficacy and less psychological distress (Alat et al., 2023; Haywood & Mason, 2022; Sigurvinsdottir et al., 2020).

Behavior is influenced by social norms, which are shaped by societal expectations and the desire to conform (Bond et al., 2012; Cialdini & Goldstein, 2004; Dickie et al., 2018; Kim et al., 2015; Legros & Cislaghi, 2020). Social norms comprise descriptive norms, which describe typical behaviors, and injunctive norms, which specify approved or disapproved behaviors in particular contexts (Neville et al., 2021). During the COVID-19 pandemic, global adoption of new norms, including mask-wearing and hand hygiene, emerged as crucial in virus containment (Fisher et al., 2020; Goldberg et al., 2020; Gouin et al., 2020; Liu et al., 2022; Neville et al., 2021; Rudert & Janke, 2022; Young & Goldstein, 2021).

The present study aimed to understand how health literacy, IHLOC, and social norms influence intentions to adhere to physical distancing during the COVID-19 pandemic. A model incorporating these factors, alongside self-efficacy and perceptions of benefits and barriers, was tested to understand the factors influencing adherence intentions in the Omani population. The first COVID-19 case in Oman was reported in February 2020, and by September 2020, there were 85,928 cases and 689 deaths. These numbers escalated to 114,438 cases and 1,208 deaths by the end of October 2021 (Dong et al., 2020). Physical distancing mandates were enforced in March 2020. Based on findings from previous research (see **Figure A** for the proposed pathways and **Table A** for a detailed rationale), the following hypotheses are proposed:

- Health literacy will be related to the three measures of physical distancing (positively related to perceived benefits, positively related to perceived self-efficacy, and negatively related to perceived barriers).
- IHLOC will be positively associated with three measures of physical distancing (positively related to perceived benefits, positively related to perceived self-efficacy, and negatively related to perceived barriers).
- Both descriptive (a) and injunctive (b) norms will be associated with three measures of physical distancing (positively related to perceived benefits, positively related to perceived self-efficacy, and negatively related to perceived barriers).

**Figure A.**
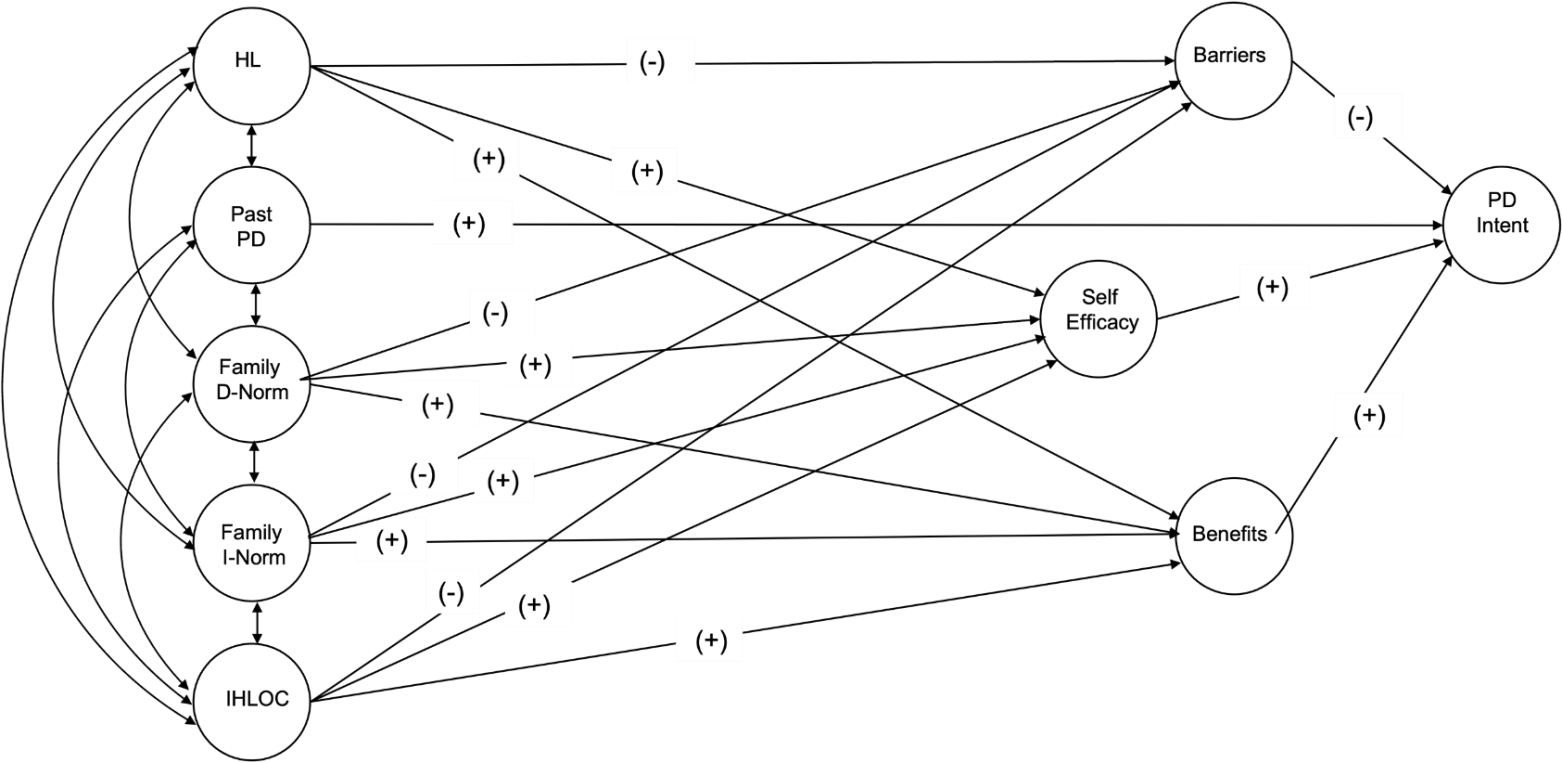
Physical Distancing Intentions Proposed Model *Note. *HL = Health Literacy; Past PD = Past physical distancing; Family D-Norm = Family physical distancing descriptive norms; Family I-Norm = Family physical distancing injunctive norms; IHLOC = Internal Health Locus of Control; SE = Self-efficacy; Barriers = perceived barriers; Benefits. = perceived benefits; PD Intent = Physical distancing intentions. For more information regarding the rationale for the pathways tested please see Table A.

**Table A:**
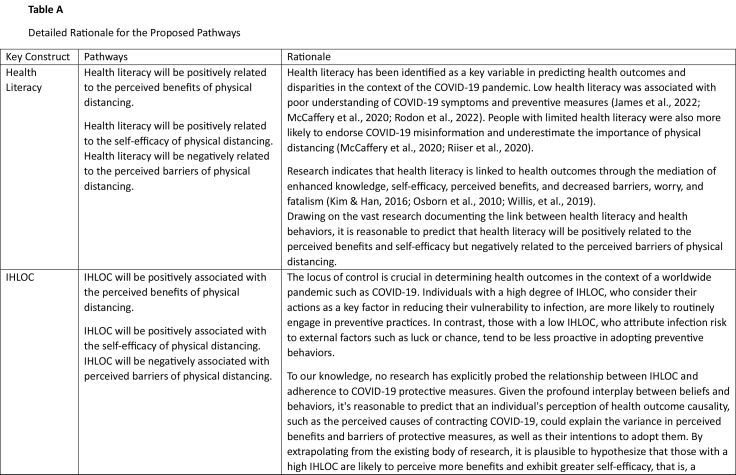
Detailed Rationale for the Proposed Pathways

Key Construct	Pathways	Rationale
Health Literacy	Health literacy will be positively related to the perceived benefits of physical distancing.	Health literacy has been identified as a key variable in predicting health outcomes and disparities in the context of the COVID-19 pandemic. Low health literacy was associated with poor understanding of COVID-19 symptoms and preventive measures (James et al., 2022; McCaffery et al., 2020; Rodon et al., 2022). People with limited health literacy were also more likely to endorse COVID-19 misinformation and underestimate the importance of physical distancing (McCaffery et al., 2020; Riiser et al., 2020).
	Health literacy will be positively related to the self-efficacy of physical distancing. Health literacy will be negatively related to the perceived barriers of physical distancing.	Research indicates that health literacy is linked to health outcomes through the mediation of enhanced knowledge, self-efficacy, perceived benefits, and decreased barriers, worry, and fatalism (Kim & Han, 2016; Osborn et al., 2010; Willis, et al., 2019). Drawing on the vast research documenting the link between health literacy and health behaviors, it is reasonable to predict that health literacy will be positively related to the perceived benefits and self-efficacy but negatively related to the perceived barriers of physical distancing.

IHLOC	IHLOC will be positively associated with the perceived benefits of physical distancing.	The locus of control is crucial in determining health outcomes in the context of a worldwide pandemic such as COVID-19. Individuals with a high degree of IHLOC, who consider their actions as a key factor in reducing their vulnerability to infection, are more likely to routinely engage in preventive practices. In contrast, those with a low IHLOC, who attribute infection risk to external factors such as luck or chance, tend to be less proactive in adopting preventive behaviors.
	IHLOC will be positively associated with the self-efficacy of physical distancing. IHLOC will be negatively associated with perceived barriers of physical distancing.	To our knowledge, no research has explicitly probed the relationship between IHLOC and adherence to COVID-19 protective measures. Given the profound interplay between beliefs and behaviors, it's reasonable to predict that an individual's perception of health outcome causality, such as the perceived causes of contracting COVID-19, could explain the variance in perceived benefits and barriers of protective measures, as well as their intentions to adopt them. By extrapolating from the existing body of research, it is plausible to hypothesize that those with a high IHLOC are likely to perceive more benefits and exhibit greater self-efficacy, that is, a

**Table:**
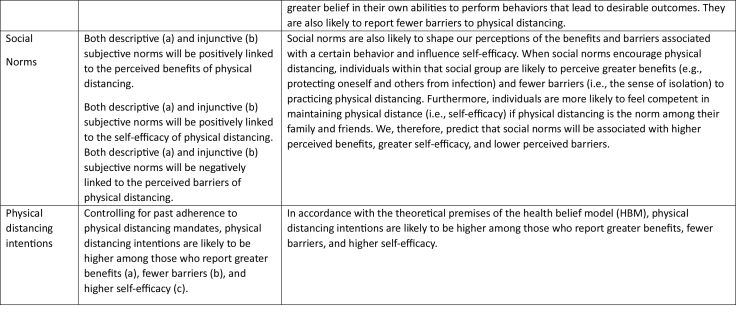


		greater belief in their own abilities to perform behaviors that lead to desirable outcomes. They are also likely to report fewer barriers to physical distancing.

Social Norms	Both descriptive (a) and injunctive (b) subjective norms will be positively linked to the perceived benefits of physical distancing.	Social norms are also likely to shape our perceptions of the benefits and barriers associated with a certain behavior and influence self-efficacy. When social norms encourage physical distancing, individuals within that social group are likely to perceive greater benefits (e.g., protecting oneself and others from infection) and fewer barriers (i.e., the sense of isolation) to practicing physical distancing. Furthermore, individuals are more likely to feel competent in maintaining physical distance (i.e., self-efficacy) if physical distancing is the norm among their family and friends. We, therefore, predict that social norms will be associated with higher perceived benefits, greater self-efficacy, and lower perceived barriers.
	Both descriptive (a) and injunctive (b) subjective norms will be positively linked to the self-efficacy of physical distancing. Both descriptive (a) and injunctive (b) subjective norms will be negatively linked to the perceived barriers of physical distancing.	

Physical distancing intentions	Controlling for past adherence to physical distancing mandates, physical distancing intentions are likely to be higher among those who report greater benefits (a), fewer barriers (b), and higher self-efficacy (c).	In accordance with the theoretical premises of the health belief model (HBM), physical distancing intentions are likely to be higher among those who report greater benefits, fewer barriers, and higher self-efficacy.

## Methods

### Participants and Procedure

This cross-sectional study used an online survey design. Ethical approval was secured from the university research department. From September 2020 to October 2020, before vaccine availability, participants were recruited via social media platforms (e.g., WhatsApp, Twitter, Instagram). They consented and responded to the Qualtrics survey, available in both English and Arabic (validated by a translator). Upon completion, they had the option to enter a cash prize drawing.

### Measures

This study used an adapted version of the COVID-19 behavioral insights survey developed by the World Health Organization (WHO). The survey included the measures listed below.

***Demographics.*** Participants indicated their age, gender, educational qualification, employment status, and COVID-19 infection. Of these factors, only gender showed a statistically significant difference in relation to the study variables (see **Table 1**).

**Table 1:**
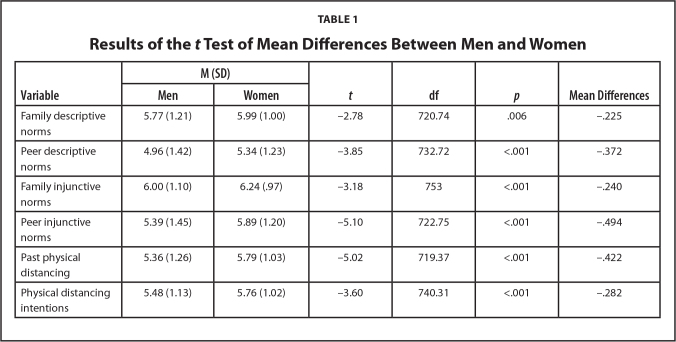
Results of the *t* Test of Mean Differences Betw een Men and Women

**Variable**	**M (SD)**		***t***	**df**	***p***	**Mean Differences**
	**Men**	**Women**				
Family descriptive norms	5.77 (1.21)	5.99 (1.00)	−2.78	720.74	.006	−.225
Peer descriptive norms	4.96 (1.42)	5.34 (1.23)	−3.85	732.72	<.001	−.372
Family injunctive norms	6.00 (1.10)	6.24 (.97)	−3.18	753	<.001	−.240
Peer injunctive norms	5.39 (1.45)	5.89 (1.20)	−5.10	722.75	<.001	−.494
Past physical distancing	5.36 (1.26)	5.79 (1.03)	−5.02	719.37	<.001	−.422
Physical distancing intentions	5.48 (1.13)	5.76 (1.02)	−3.60	740.31	<.001	−.282

***Health literacy.*** Participants rated their ability to access, understand, appraise, and apply COVID-19 information and guidelines using nine items on a seven-point Likert scale, ranging from 1 (*extremely difficult*) to 7 (*extremely easy*). This scale is an adaptation suited for diverse contexts and is based on the European Health Literacy Survey Questionnaire (HLS-EU-Q) developed and validated by Sørensen et al., (2013). The WHO's behavioral insights survey employed a modified version of this tool.

***Physical distancing behavioral intention.*** Participants rated their intention to adhere to physical distancing mandates using eight items, such as “How likely are you to avoid family visits?” on a seven-point Likert scale from 1 (*extremely unlikely*) to 7 (*extremely likely*).

***Self-efficacy.*** Participants rated their confidence in performing physical distancing using two items (e.g., “I am confident that I have the ability to participate in physical distancing measures”) on a seven-point Likert scale from 1 (*strongly disagree*) to 7 (*strongly agree*).

In addition to the scales provided in the WHO's behavioral insights survey, the following measures were added:

***Past physical distancing.*** Participants rated their past physical distancing over the last four weeks on a scale from 1 (never) to 7 (every time), using the same eight items as in the intentions measure. Higher scores indicated more distancing. Past distancing served as a control variable for future adherence intentions.

***Social norms.*** Norms regarding physical distancing were assessed with two primary reference groups: (1) family and (2) friends/co-workers/colleagues. Descriptive norms, or perceived adherence to physical distancing, were evaluated using three items for each reference group (e.g., “My family/friends/co-workers/colleagues avoid social visits and gatherings”). Injunctive norms, which focus on beliefs about perceived approval, were measured through similar items (e.g., “My family/friends/coworkers/colleagues believe I should avoid social visits and gatherings”).

***IHLOC.*** IHLOC was assessed with six items created by Wallston et al. (1978), using statements like “If I get sick, it is my own behavior that determines how quickly I recover.”

***Perceived benefits.*** Benefits were measured using three items (e.g., “I will be less vulnerable to a COVID-19 infection if I adopt preventive physical distancing measures”). This scale is a modified version of one developed and validated by Cheng and Ng (2006).

Participants rated the social norms, IHLOC, and perceived benefits statements on a seven-point Likert scale from 1 (*strongly disagree*) to 7 (*strongly agree*).

***Perceived barriers.*** Participants rated ten potential obstacles to physical distancing (e.g., refraining from family visits) on a seven-point Likert scale ranging from 1 (*very simple*) to 7 (*extremely difficult*). The scale was adapted from Cheng and Ng (2006).

## Data Analysis

Initial correlational analyses were performed in SPSS (Version 28) to explore relationships between variables, and ANOVA (analysis of variance) was used to examine gender differences. The model was tested through structural equation modeling (SEM) in Mplus version 7.2, using maximum likelihood parameter estimates with standard errors and a mean-adjusted chi-square test statistic that are robust to non-normality (MLM) (Muthén & Muthén, 2012). Regression effects are reported as standardized beta weights (β) and *p* values. Mediation was assessed using bias-corrected bootstrap 95% confidence intervals [CI] with 2,000 bootstrap samples. Good model fit indicators include lower and nonsignificant chi-squared values; comparative fit index (CFI) and Tucker Lewis Index (TLI) >0.90; root mean square error of approximation (RMSEA) ≤0.06; and standardized root mean square residual (SRMR) <.08 (Byrne, 2013). The sample was gender-weighted to correct any potential bias, thus significantly improving the model fit.

## Results

A total of 759 participants, between ages 18 and 63 years (*M* = 29.13, standard deviation [*SD*] = 8.33), completed the survey. Of these, 68.5% were women (*n* = 520) and 93.7% identified as Omani. The detailed sample characteristics are in **Table 2**, and Cronbach's alpha, descriptive statistics, and zero-order correlations for the scales are shown in **Table 3**.

**Table 2:**
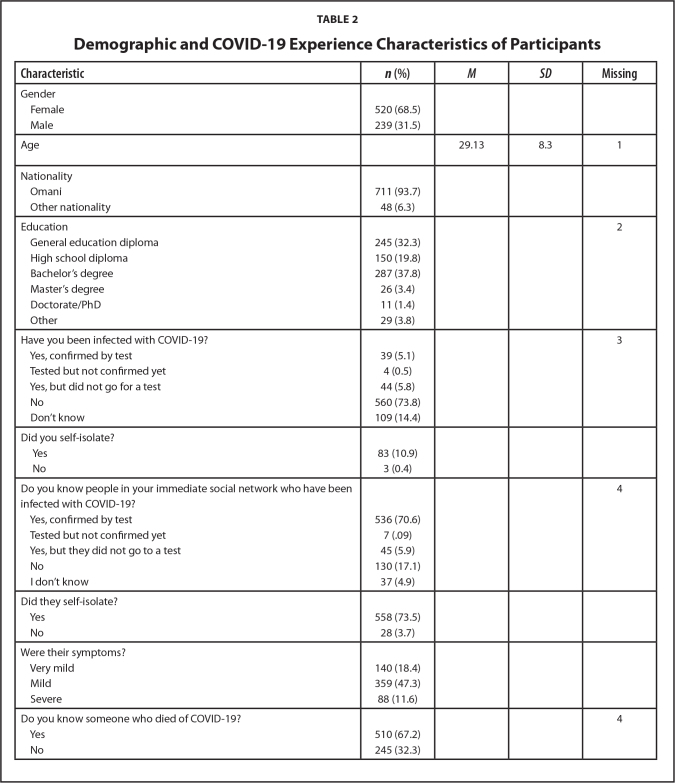
Demographic and COVID-19 Experience Characteristics of Participants

**Characteristic**	***n* (%)**	***M***	***SD***	**Missing**

Gender				
Female	520 (68.5)			
Male	239 (31.5)			

Age		29.13	8.3	1

Nationality				
Omani	711 (93.7)			
Other nationality	48 (6.3)			

Education				2
General education diploma	245 (32.3)			
High school diploma	150 (19.8)			
Bachelor's degree	287 (37.8)			
Master's degree	26 (3.4)			
Doctorate/PhD	11 (1.4)			
Other	29 (3.8)			

Have you been infected with COVID-19?				3
Yes, confirmed by test	39 (5.1)			
Tested but not confirmed yet	4 (0.5)			
Yes, but did not go for a test	44 (5.8)			
No	560 (73.8)			
Don't know	109 (14.4)			

Did you self-isolate?				
Yes	83 (10.9)			
No	3 (0.4)			

Do you know people in your immediate social network who have been infected with COVID-19?				4
Yes, confirmed by test	536 (70.6)			
Tested but not confirmed yet	7 (.09)			
Yes, but they did not go to a test	45 (5.9)			
No	130 (17.1)			
I don't know	37 (4.9)			

Did they self-isolate?				
Yes	558 (73.5)			
No	28 (3.7)			

Were their symptoms?				
Very mild	140 (18.4)			
Mild	359 (47.3)			
Severe	88 (11.6)			

Do you know someone who died of COVID-19?				4
Yes	510 (67.2)			
No	245 (32.3)			

**Table 3:**
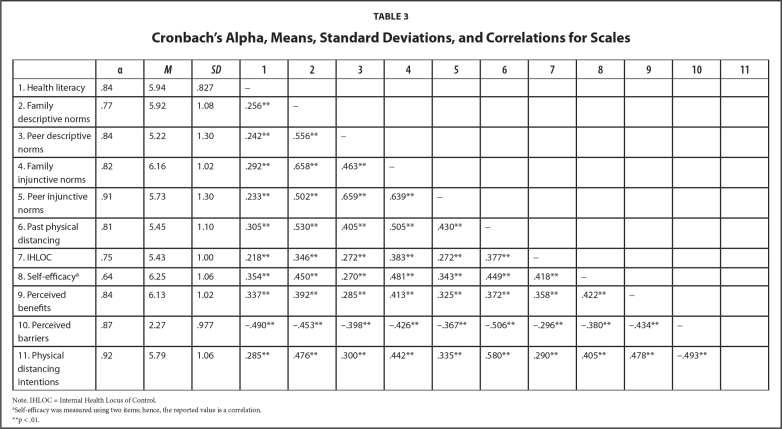
Cronbach's Alpha, Means, Standard Deviations, and Correlations for Scales

	**α**	***M***	***SD***	**1**	**2**	**3**	**4**	**5**	**6**	**7**	**8**	**9**	**10**	**11**
1. Health literacy	.84	5.94	.827	-										
2. Family descriptive norms	.77	5.92	1.08	.256^**^	-									
3. Peer descriptive norms	.84	5.22	1.30	.242^**^	.556^**^	-								
4. Family injunctive norms	.82	6.16	1.02	.292^**^	.658^**^	.463^**^	-							
*5. *Peer injunctive norms	.91	5.73	1.30	.233^**^	.502^**^	.659^**^	.639^**^	-						
6. Past physical distancing	.81	5.45	1.10	.305^**^	.530^**^	.405^**^	.505^**^	.430^**^	-					
7. IHLOC	.75	5.43	1.00	.218^**^	.346^**^	.272^**^	.383^**^	.272^**^	.377^**^	-				
8. Self-efficacy^a^	.64	6.25	1.06	.354^**^	.450^**^	.270^**^	.481^**^	.343^**^	.449^**^	.418^**^	-			
9. Perceived benefits	.84	6.13	1.02	.337^**^	.392^**^	.285^**^	.413^**^	.325^**^	.372^**^	.358^**^	.422^**^	-		
10. Perceived barriers	.87	2.27	.977	−.490^**^	−.453^**^	−.398^**^	−.426^**^	−.367^**^	−.506^**^	−.296^**^	−.380^**^	−.434^**^	-	
11. Physical distancing intentions	.92	5.79	1.06	.285^**^	.476^**^	.300^**^	.442^**^	.335^**^	.580^**^	.290^**^	.405^**^	.478^**^	−.493^**^	

Note. IHLOC = Internal Health Locus of Control.

a Self-efficacy was measured using two items; hence, the reported value is a correlation.

** p < .01.

An ANOVA test comparing means revealed gender differences in some study variables. Specifically, women reported higher physical distancing norms, past adherence, and intentions than men (see **Table 1**).

**Figure 1.**
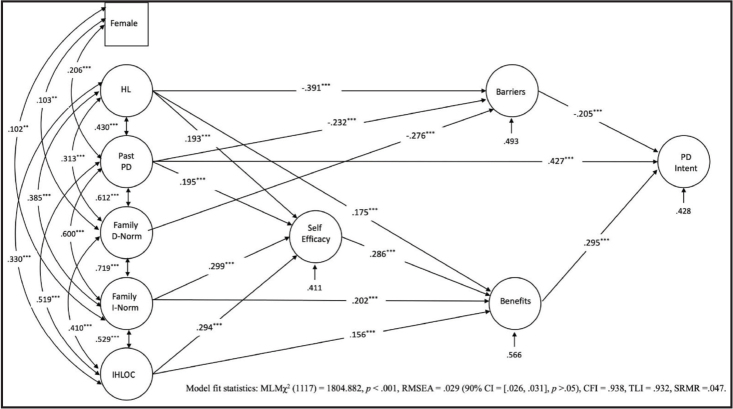
The physical distancing intentions of the structural equation model.

The SEM analysis included all significant independent variables associated with physical distancing intentions following correlational and univariate analyses. Gender was added as a control variable due to its significant correlation with the variables. The proposed model was tested and modified in Mplus, resulting in the model shown in **Figure 1**. This model affirmed the ANOVA findings, substantiating the positive association between the female gender and previous compliance with physical distancing, referred to as “past PD” (*r* = .206, *p* < .001), as well as family descriptive norms (*r* = .103, *p* = .003) and injunctive norms (*r* = .102, *p* = .002).

As predicted in H1, health literacy was linked with more perceived benefits (*β* = .175, *p* = .001), greater self-efficacy (β =.193, *p* < .001), and fewer perceived barriers (*β* = −.391, *p* < .001). It was also positively associated with past adherence to physical distancing (*r* = .430, *p* < .001). Furthermore, H2 was partially supported. IHLOC was positively associated with greater perceived benefits (β =.156, *p* = .007) and self-efficacy (β =.294, *p* < .001), but was not significantly associated with barriers.

The model explored and tested relationships between norms, perceived benefits and barriers, and intentions toward physical distancing. Although peer descriptive and injunctive norms were significantly related to gender (refer to **Table 1**), they were not included in the model as they did not predict the outcome variables. On the other hand, family descriptive norms were significantly associated with fewer perceived barriers (*β* = −.276, *p* < .001), thereby confirming the proposed pathway in hypothesis H3. However, there were no significant associations between descriptive norms and the perceived benefits and self-efficacy of physical distancing. Injunctive norms, in contrast, were associated with more perceived benefits (*β* = .202, *p* = .001) and greater self-efficacy (*β* = .299, *p* < .001) but were not significantly linked with perceived barriers, contrary to the proposed pathway in hypothesis H3.

The model further depicts that the intentions of compliance with the physical distancing mandates were significantly associated with past physical distancing (*β* = .427, *p* < .001) and perceived barriers (β = −.205, *p* < .001) and benefits (*β* = .295, *p* < .001). Although self-efficacy did not directly predict physical distancing intentions, it was significantly associated with more perceived benefits (β =.286, *p* < .001). Post-hoc mediation analysis revealed that perceived benefits mediated the relationship between self-efficacy and intentions (*β* = .084, 95% CI = [.027, .170], *p* = .019) and between IHLOC and intentions (*β* = .071, 95% CI = [.012, .105], *p* = .005).

The post-hoc mediation analysis also revealed several other small yet significant indirect effects (see **Table 4**). Perceived barriers mediated the relationship between descriptive norms and physical distancing intentions (*β* = .057, 95% CI = [.015, .122], *p* < .041). Perceived benefits mediated the relationship between injunctive norms and physical distancing intentions (*β* = .060, 95% CI = [.017, .133], *p* < .035). The relationship between health literacy and intentions was mediated by both perceived benefits (*β* = .052, 95% CI = [.019, .096], *p* = .009) and barriers (*β* = .080, 95% CI = [.031, .140], *p* = .004). The overall model accounted for over 57% of the variance in physical distancing intentions and demonstrated a good fit, MLMχ2 (1117) = 1804.882, *p* < .001, RMS*EA* = .029 (90% CI = [.026, .031], *p* > .05), C*FI* = .938, T*LI* = .932, SR*MR* = .047.

**Table 4:**
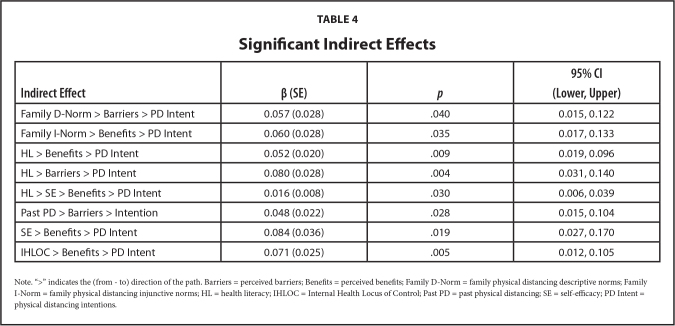
Significant Indirect Effects

**Indirect Effect**	**β (SE)**	***p***	**95% CI (Lower, Upper)**
Family D-Norm > Barriers > PD Intent	0.057 (0.028)	.040	0.015, 0.122
Family I-Norm > Benefits > PD Intent	0.060 (0.028)	.035	0.017, 0.133
HL > Benefits > PD Intent	0.052 (0.020)	.009	0.019, 0.096
HL > Barriers > PD Intent	0.080 (0.028)	.004	0.031, 0.140
HL > SE > Benefits > PD Intent	0.016 (0.008)	.030	0.006, 0.039
Past PD > Barriers > Intention	0.048 (0.022)	.028	0.015, 0.104
SE > Benefits > PD Intent	0.084 (0.036)	.019	0.027, 0.170
IHLOC > Benefits > PD Intent	0.071 (0.025)	.005	0.012, 0.105

Note. “>” indicates the (from - to) direction of the path. Barriers = perceived barriers; Benefits = perceived benefits; Family D-Norm = family physical distancing descriptive norms; Family I-Norm = family physical distancing injunctive norms; HL = health literacy; IHLOC = Internal Health Locus of Control; Past PD = past physical distancing; SE = self-efficacy; PD Intent = physical distancing intentions.

## Discussion

This study examined the factors associated with the intentions to adhere to physical distancing mandates in Oman. Results indicated that health literacy is indirectly linked with intentions to adhere to distancing mandates. As anticipated, participants with higher health literacy perceived more benefits and fewer barriers to distancing and exhibited greater confidence in their adherence. Both perceived benefits and barriers mediated the relationship between health literacy and behavioral intentions, corroborating prior research (Goto et al., 2019; Oldach & Katz, 2014; Rudd, 2019; Sarma et al., 2019). This study confirms that even among participants with notably high health literacy levels (*M* = 5.94; *SD* = 0.827), health literacy significantly influenced perceptions of benefits, barriers, and self-efficacy related to distancing intentions. This suggests that improving health literacy might strengthen physical distancing adherence, even in well-informed individuals.

The findings of this study further emphasize the pivotal role of IHLOC in shaping intentions to adhere to physical distancing. Consistent with prior research (Bennett et al., 2017; Marr & Wilcox, 2015; Steptoe & Wardle, 2001), IHLOC was positively correlated with self-efficacy and perceived benefits. This link indicates that strategies highlighting personal accountability for contracting and transmitting the virus could be successful. Attributional retraining strategies, for instance, can enhance confidence in complying with distancing measures and amplify the perceived benefits of such behaviors (Parker et al., 2022). Additionally, the positive relationships between IHLOC, health literacy, family norms, and past adherence imply that family support and previous adherence can enhance IHLOC. This synergy suggests that interventions that strengthen individual accountability, family support, and health literacy could be instrumental in sustaining physical distancing.

Moreover, the results highlight the significant role of family norms in shaping adherence intentions and illuminate the distinct effects of descriptive and injunctive norms. Descriptive norms, focusing on accuracy and efficiency, dictate how household members interact and set guidelines to reduce risks, such as avoiding crowds and keeping distance. Injunctive norms, on the other hand, are linked with enhanced perceived benefits. They frequently emphasize what one “should do” to reap benefits like lower vulnerability to COVID-19 (Jacobson et al., 2011). Due to their cognitive and psychological underpinnings, descriptive and injunctive norms can influence health behavior differently (Jacobson et al., 2011; Meisel et al., 2015). Future research should further investigate the differential effects of descriptive and injunctive norms on health behavior. The results could lead to more nuanced and focused public health interventions and help leverage each's capabilities to create more comprehensive and contextually relevant interventions.

This study sheds light on several important implications. First, health literacy, often sidelined in Oman and other developing countries, is fundamental for adhering to physical distancing and overall pandemic control. Thus, Omani public health policy should elevate health literacy as essential for driving health behaviors. Further, the prominence of IHOLC in boosting self-efficacy and perceived benefits highlights the value of emphasizing individual control over health outcomes, particularly during health crises like COVID-19. This perspective could instigate more robust health responses. Finally, the indirect link between family norms and distancing intentions underscores the efficacy of family-and community-centric interventions in enhancing adherence to protective measures, especially during pandemics.

## Study Limitations

This study has several limitations that should be noted when interpreting the findings. First, the results are correlational and do not imply causation. Second, reliance on self-reported data may have introduced cognitive and social desirability biases. Third, the survey lasted 2 months, during which COVID-19 cases changed. This changing landscape may have affected respondents' opinions. Fourth, the WHO's behavioral insight survey was designed to be repeated to track changes. However, this study used it as a one-time survey due to budget restrictions, making tracking behavior and attitude changes challenging. Longitudinal or repeated cross-sectional surveys may yield additional nuanced insights. Fifth, the significance of gender in our analyses was not anticipated. Future research is warranted to ascertain the generalizability of this observation.

Finally, participants exhibited notably high health literacy. This could stem from recruitment through social media, attracting those proficient with online health information. The surge in health awareness during COVID-19 might also have played a role. This recruitment approach and the pandemic's information-rich climate could have skewed our sample toward individuals with superior health literacy and social media use. This poses potential generalizability issues, especially for populations with lower health literacy or limited social media exposure. To obtain a more inclusive and representative sample, it is recommended that future research should explore various recruitment methods.

## Conclusion

Results show that health literacy and IHLOC significantly influence perceived benefits and self-efficacy in physical distancing. While family descriptive norms reduce perceived barriers, injunctive norms boost perceived benefits and self-efficacy. Adherence intentions to distancing mandates are significantly associated with past behavior and perceived barriers and benefits. These findings are vital for decision-makers, indicating that prioritizing health literacy, IHLOC, and family values can improve adherence to distancing measures.
